# Border tissue morphology is associated with the pattern of visual field progression in open-angle glaucoma

**DOI:** 10.1038/s41598-022-16186-3

**Published:** 2022-07-14

**Authors:** Hyun Joo Kee, Jong Chul Han, Eui Do Song, Eui Jun Choi, Dong Ook Son, Eun Jung Lee, Yoon Kyoung Jang, Changwon Kee

**Affiliations:** 1 Department of Ophthalmology, Samsung Medical Center, Sungkyunkwan University School of Medicine, Seoul, 06351 Korea; 2 Nunemiso Eye Center, Seoul, 06241 Korea; 3 Department of Medical Device, Management and Research, SAIHST, Sungkyunkwan University, Seoul, 06351 Korea; 4 Department of Statistics and Data Science, Yonsei University, Seoul, 03722 Korea

**Keywords:** Optic nerve diseases, Vision disorders

## Abstract

The etiology of open-angle glaucoma (OAG) is yet unclear. This study investigated possible risk factors, such as the morphology of the border tissue that affect the pattern of visual field (VF) progression in eyes with OAG. 166 eyes of 166 OAG patients with an externally oblique border tissue (EOBT) at least in one direction were included. EOBT was obtained by analyzing enhanced depth imaging spectral-domain optical coherence tomography images. A pointwise linear regression was used to determine VF progression by measuring the deterioration rate of each point in the VF. The odds ratio of VF progression for each risk factor was estimated using logistic regression analysis. Seventy (42.2%) eyes showed VF deterioration. In multivariate analysis, longer follow-up period, higher baseline intraocular pressure (IOP), lower mean ocular perfusion pressure (MOPP), and smaller angular location of the longest EOBT were associated with VF progression (all *p* values were below 0.05). In the multivariate analysis, the location of the longest EOBT was significantly associated with inferior (*p* = 0.002) and central (*p* = 0.017) VF progression. In conclusion, VF progression pattern in OAG eyes is associated with the location of the longest EOBT as well as other known risk factors.

## Introduction

Open-angle glaucoma (OAG) is a progressive optic neuropathy with corresponding glaucomatous visual field (VF) defects^1^. Glaucoma has various types of VF defect patterns including arcuate defects, nasal steps, and other patterns associated with retinal nerve fiber layer (RNFL) defects. Although glaucoma patients can have good visual acuity, many have difficulties in activities of daily life according to VF defect pattern^2–4^. Thus, it is important to predict the final VF pattern considering that VF pattern can affect quality of life^5,6^.

A few studies have reported possible risk factors associated with specific VF defect patterns, and various risk factors for glaucomatous VF progression have been introduced^7^. Several vascular factors such as hypotension, migraine, Raynaud’s phenomenon, cardiovascular problems, and unstable mean ocular perfusion pressure (MOPP) are known to be associated with central or paracentral scotoma^8–10^. Meanwhile, ONH and surrounding structures such as optic nerve head (ONH) tilt or beta-zone parapapillary atrophy have also been reported to be associated with specific VF progression patterns in glaucoma^11,12^. As detailed features of ONH have become measurable in this era of optical coherence tomography (OCT), several studies have shown that deep ONH structures such as the lamina cribrosa or border tissue of Elschnig are related to VF defect patterns in glaucomatous eyes^13,14^. In our previous studies, the longest externally oblique border tissue (EOBT) was related to the direction of the RNFL defect, worse VF defects, and glaucoma-associated findings such as lamina cribrosa defects or choroidal microvascular dropout in OAG eyes^14–16^.

However, there has been no long-term longitudinal study demonstrating the influence of border tissue morphology on the pattern of VF progression. In the present study, we hypothesized that several factors, including the morphology of the border tissue and other risk factors, may simultaneously affect the pattern of VF progression in OAG eyes. To investigate this hypothesis, we investigated possible factors that could influence the pattern of VF progression in treated OAG patients who underwent long-term observation.

## Results

A total of 166 eyes of 166 patients with OAG were included in the analysis. Interobserver agreement of length and location of the longest EOBT were 0.969 (0.960–0.978) and 0.963 (0.948–0.978), respectively. Mean follow-up period was 11.7 ± 4.7 years. During the follow-up period, the mean number of VF measurements was 11.6 ± 4.6. Baseline age was 49.5 ± 12.6 years and 98 patients (59.0%) were male. The number of intraocular pressure (IOP)-lowering medications at the first visit and the mean number of IOP-lowering medications were 0.5 ± 0.8 and 0.7 ± 0.9, respectively. Central corneal thickness (CCT) and axial length were 530.3 ± 35.8 um and 25.1 ± 1.5 mm, respectively. Baseline IOP and mean follow-up IOP were 17.4 ± 4.6 mmHg and 15.9 ± 3.0 mmHg, respectively. Mean systolic blood pressure (SBP), mean diastolic blood pressure (DBP), and mean MOPP were 124.3 ± 13.5 mmHg, 74.4 ± 11.1 mmHg, and 50.9 ± 7.1 mmHg, respectively. Average maximum EOBT length was 425.3 ± 286.1 μm and the location of maximum EOBT length was 23.1 ± 38.7 degrees. Average baseline mean deviation (MD) and pattern standard deviation (PSD) were − 3.4 ± 3.2 dB and 5.6 ± 3.9 dB, respectively, and average final MD and PSD were − 7.1 ± 5.6 dB and 8.8 ± 4.3 dB, respectively. Overall, 70 eyes (42.2%) showed significant progression in the VF test. VF progression only in the superior hemifield was found in 33 patients (19.9%) and that only in the inferior hemifield was found in 21 patients (12.7%). Sixteen patients (9.6%) had VF progression only in the central area (Table 1).

**Table 1:** Participant demographics and clinical characteristics.

Variables	Description
Patients (n)	166
Follow-up duration (yrs)	11.7 ± 4.7
VF measurements (n)	11.6 ± 4.6
Age at the first visit (yrs)	49.5 ± 12.6
Male sex (n, %)	98 (59.0%)
Number of medications at the first visit (n)	0.5 ± 0.8
Mean number of IOP-lowering medications (n)	0.7 ± 0.9
Central corneal thickness (μm)	530.3 ± 35.8
Axial length (mm)	25.1 ± 1.5
**IOP, BP and MOPP**	
Baseline IOP (mmHg)	17.4 ± 4.6
Mean follow-up IOP (mmHg)	15.9 ± 3.0
Mean SBP (mmHg)	124.3 ± 13.5
Mean DBP (mmHg)	74.4 ± 11.1
Mean MOPP (mmHg)	50.9 ± 7.1
**Border tissue characteristics**	
Maximum EOBT length (μm)	425.3 ± 286.1
Location of maximum EOBT (°)	23.1 ± 38.7
**Baseline VF examination**	
MD (dB)	− 3.4 ± 3.2
PSD (dB)	5.6 ± 3.9
**Final VF examination**	
MD (dB)	− 7.1 ± 5.6
PSD (dB)	8.8 ± 4.3
**Progression cases**	
Overall progression cases (n, %)	70 (42.2)
Superior hemifield (n, %)	33 (19.9)
Inferior hemifield (n, %)	21 (12.7)
Central area (n, %)	55 (36.4)

*DBP* diastolic blood pressure, *EOBT* externally oblique border tissue, *IOP* intraocular pressure, *MD* mean deviation, *MOPP* mean ocular perfusion pressure, *PSD* pattern standard deviation, *SBP* systolic blood pressure, *VF* visual field.

Data are presented as the means ± standard deviations unless otherwise indicated.

**Figure 1:**
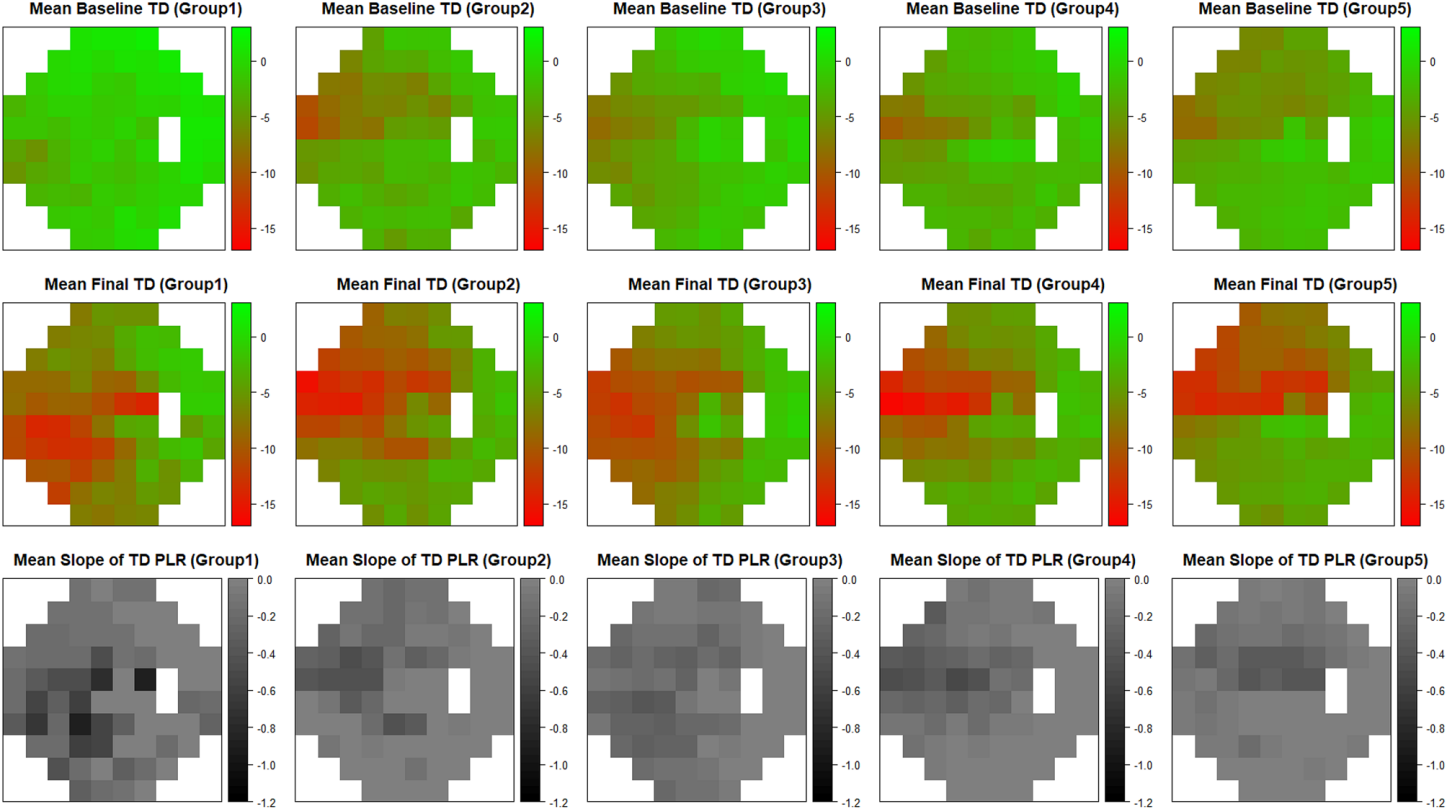
Relationship between the location of the maximum externally oblique border tissue (EOBT) and visual field (VF) progression. Columns show the five groups classified by location of the maximum EOBT. The first and second rows compare the mean total deviation plots (dB) at first and last visit, and the gray scale plot on the bottom row indicates the average slope of the total deviation plot at each point (dB/year). Progression rates are shown in grayscale in the bottom row; the lightest sectors showed the slowest progression while the darkest sectors showed the fastest progression. At the first visit, most of the groups showed VF defect at the superior hemifield except group 1, and at the last visit, the pattern of VF defect seems to be more obvious according to the location of the maximum EOBT. VF progression was dominantly in the inferior hemifield in group 1, whereas it was dominantly in the superior hemifield in group 5. TD = total deviation, PLR = point-wise linear regression.

When patients were divided into subgroups according to the location of the longest EOBT, there were no significant differences except for maximum EOBT length, age at the first visit, axial length, and VF progression in the inferior hemifield (*p* < 0.001; *p* = 0.018; *p* = 0.001; *p* = 0.003, respectively). Groups 2, 3, and 4 were likely to have greater maximum EOBT length, a younger age, and longer axial length than groups 1 and 5. The VF progression cases in the inferior hemifield were the most in group 1 (Table 2, Fig. 1).

**Table 2:** Comparisons of variables among groups divided by the location of maximum externally oblique border tissue.

Variables	Group 1 (n = 8)	Group 2 (n = 20)	Group 3 (n = 41)	Group 4 (n = 43)	Group 5 (n = 54)	*p* value
Maximum EOBT location	** < − 45°**	**− 45° ~ − 15°**	**− 15° ~ + 15°**	** + 15° ~ + 45°**	** > + 45°**	N/A
Maximum EOBT length (μm)	213.9 ± 79.9	495.5 ± 410.5	523.9 ± 305.8	474.2 ± 247.2	317.0 ± 211.1	< 0.001*
Follow-up duration (mos)	129.4 ± 51.4	115.5 ± 40.4	147.0 ± 50.9	142.2 ± 58.6	145.1 ± 61.8	0.411*
VF measurements (n)	10.9 ± 4.3	9.3 ± 3.3	12.3 ± 4.1	12.1 ± 4.8	11.8 ± 5.1	0.167*
Age at the first visit (yrs)	58.5 ± 6.1	49.1 ± 13.6	46.1 ± 12.2	47.4 ± 12.8	52.6 ± 12.0	0.018*
Male Sex (n, %)	2 (25)	14 (70)	26 (63.4)	27 (62.8)	29 (53.7)	0.214^†^
Medication number at first visit (n)	0.4 ± 0.7	0.7 ± 1.2	0.5 ± 0.8	0.4 ± 0.7	0.4 ± 0.8	0.891*
Mean medication number (n)	0.6 ± 0.4	1.0 ± 0.9	1.1 ± 0.7	1.0 ± 0.6	0.9 ± 0.6	0.229*
Central corneal thickness (μm)	540.6 ± 36.6	539.1 ± 43.7	527.5 ± 36.5	532.5 ± 31.5	526.0 ± 35.4	0.233*
Axial length (mm)	24.5 ± 1.2	25.2 ± 1.6	25.8 ± 1.6	25.4 ± 1.3	24.5 ± 1.3	0.001*
Baseline IOP (mmHg)	15.3 ± 3.0	16.2 ± 3.4	17.8 ± 3.7	17.9 ± 6.6	17.6 ± 3.7	0.174*
Mean follow-up IOP (mmHg)	13.3 ± 2.7	14.6 ± 2.4	15.5 ± 2.8	14.9 ± 2.9	14.2 ± 3.1	0.181*
Mean MOPP (mmHg)	50.3 ± 5.8	54.5 ± 8.3	51.1 ± 7.6	51.2 ± 5.7	49.2 ± 7.2	0.228*
Baseline MD (dB)	− 1.1 ± 1.8	− 4.2 ± 4.0	− 3.0 ± 3.2	− 3.5 ± 2.9	− 3.7 ± 3.1	0.107*
Final MD (dB)	− 7.7 ± 7.2	− 8.0 ± 6.7	− 7.2 ± 6.6	− 6.9 ± 4.7	− 6.9 ± 4.7	0.983*
Superior VF Progress (n, %)	3 (37.5)	6 (30)	11 (26.8)	13 (30.2)	16 (29.6)	0.977^†^
Inferior VF Progress (n, %)	4 (50)	3 (15)	12 (29.3)	14 (32.6)	4 (7.4)	**0.003**^†^
Central VF Progress (n, %)	4 (50)	6 (30)	13 (31.7)	17 (39.5)	15 (27.8)	0.608^†^

*EOBT* externally oblique border tissue, *IOP* intraocular pressure, *MD* mean deviation, *MOPP* mean ocular perfusion pressure, *VF* visual field.

Data are presented as the means ± standard deviations unless otherwise indicated.

*Kruskal–Wallis test.

^†^Fisher’s Exact test.

### Factors associated with visual field progression locations

In univariate analysis, only a longer follow-up period was associated with the presence of overall VF progression in OAG eyes (*p* < 0.001). However, in multivariate analysis, overall VF progression was associated with a longer follow-up period, higher baseline IOP, lower mean IOP, lower MOPP, and smaller longest EOBT angular location (*p* = 0.002; *p* = 0.024; *p* = 0.026; *p* = 0.005; *p* = 0.007, respectively) (Table 3).

**Table 3:** Univariate and multivariate logistic regression analyses of visual field progression.

Risk factors	Overall visual field progression 70 (42.2%)			
	Univariate analysis		Multivariate analysis	
	OR (95% CI)	*p* value	OR (95% CI)	*p* value
Follow-up period (yrs)	1.142 (1.065, 1.231)	** < 0.001**	1.141 (1.052, 1.245)	**0.002**
Age at the first visit (yrs)	0.998 (0.973, 1.023)	0.850		
Male sex	0.789 (0.421, 1.476)	0.458		
Mean number of medications	1.035 (0.640, 1.664)	0.887		
Baseline IOP (mmHg)	1.063 (0.992, 1.146)	0.093	1.232 (1.036, 1.490)	**0.024**
Mean IOP (mmHg)	1.007 (0.907, 1.117)	0.902	0.746 (0.572, 0.961)	**0.026**
Central corneal thickness (μm)	0.992 (0.983, 1.001)	0.076	0.991 (0.981, 1.002)	0.112
Mean MOPP (mmHg)	0.957 (0.913, 1.001)	0.056	0.925 (0.874, 0.975)	**0.005**
Axial length (mm)	1.161 (0.943, 1.437)	0.161		
Baseline MD (dB)	0.968 (0.878, 1.066)	0.504		
Maximum EOBT location (°)	0.995 (0.987, 1.003)	0.209	0.987 (0.977, 0.996)	**0.007**
Maximum EOBT length (μm)	1.001 (0.999, 1.002)	0.397		

*CI* confidence interval, *EOBT* externally oblique border tissue, *IOP* intraocular pressure, *MD* mean deviation, *MOPP* mean ocular perfusion pressure.

The best multiple logistic regression model was chosen based on the Akaike Information Criterion. Factors with statistical significance are marked in bold.

Univariate risk factors associated with VF progression in the superior hemifield were longer follow-up period (*p* = 0.014) and lower mean MOPP (*p* = 0.041). Only risk factor associated with VF progression in the superior hemifield based on multivariate analysis was lower mean MOPP (*p* = 0.014) (Table 4).

**Table 4:** Risk factors associated with visual field progression in the superior hemifield.

Risk factors	Superior visual field progression 33 (25.6%)			
	Univariate analysis		Multivariate analysis	
	OR (95% CI)	*p* value	OR (95% CI)	*p* value
Follow-up period (yrs)	1.122 (1.025, 1.232)	**0.014**	1.107 (0.999, 1.231)	0.054
Age at the first visit (yrs)	0.998 (0.966, 1.031)	0.905		
Male sex	1.097 (0.488, 2.545)	0.824		
Mean number of medications	0.735 (0.371, 1.362)	0.348		
Baseline IOP (mmHg)	1.082 (0.986, 1.194)	0.099	1.210 (0.991, 1.507)	0.071
Mean IOP (mmHg)	1.024 (0.887, 1.179)	0.743	0.799 (0.584, 1.076)	0.145
Central corneal thickness (μm)	0.989 (0.978, 1.0004)	0.064	0.990 (0.977, 1.002)	0.115
Mean MOPP (mmHg)	0.942 (0.886, 0.996)	**0.041**	0.920 (0.858, 0.980)	**0.014**
Axial length (mm)	1.051 (0.792, 1.389)	0.727		
Baseline MD (dB)	1.036 (0.911, 1.190)	0.599		
Maximum EOBT location (°)	1.002 (0.992, 1.013)	0.677		
Maximum EOBT length (μm)	1.0002 (0.999, 1.002)	0.800		

*CI* confidence interval, *EOBT* externally oblique border tissue, *IOP* intraocular pressure, *MD* mean deviation, *MOPP* mean ocular perfusion pressure.

The best multiple logistic regression model was chosen based on the Akaike Information Criterion. Factors with statistical significance are marked in bold.

A risk factor associated with VF progression in the inferior hemifield in univariate analyses was longer follow-up period (*p* = 0.0002). In multivariate analysis, the risk factors were longer follow-up period, worse baseline MD, and smaller longest EOBT angular location (*p* = 0.0004; *p* = 0.039; *p* = 0.005, respectively) (Table 5).

**Table 5:** Risk factors associated with visual field progression in the inferior hemifield.

Risk factors	Inferior visual field progression 21 (18.6%)			
	Univariate analysis		Multivariate analysis	
	OR (95% CI)	*p* value	OR (95% CI)	*p* value
Follow-up period (yrs)	1.223 (1.105, 1.371)	**0.0002**	1.290 (1.132, 1.509)	**0.0004**
Age at the first visit (yrs)	0.983 (0.944, 1.023)	0.398		
Male sex	0.836 (0.322, 2.231)	0.714		
Mean number of medications	1.527 (0.797, 2.882)	0.191		
Baseline IOP (mmHg)	1.091 (0.991, 1.212)	0.078	1.243 (0.962, 1.672)	0.118
Mean IOP (mmHg)	1.051 (0.895, 1.225)	0.527	0.727 (0.487, 1.047)	0.098
Central corneal thickness (μm)	0.999 (0.985, 1.014)	0.921		
Mean MOPP (mmHg)	0.978 (0.912, 1.047)	0.525	0.942 (0.864, 1.023)	0.153
Axial length (mm)	1.340 (0.973, 1.866)	0.075		
Baseline MD (dB)	0.926 (0.807, 1.068)	0.279	0.831 (0.693, 0.990)	**0.039**
Maximum EOBT location (°)	0.992 (0.979, 1.004)	0.182	0.972 (0.952, 0.990)	**0.005**
Maximum EOBT length (μm)	1.001 (0.999, 1.002)	0.289		

*CI* confidence interval, *EOBT* externally oblique border tissue, *IOP* intraocular pressure, *MD* mean deviation, *MOPP* mean ocular perfusion pressure.

The best multiple logistic regression model was chosen based on the Akaike Information Criterion. Factors with statistical significance are marked in bold.

Risk factors associated with central VF progression were longer follow-up period and greater baseline IOP in univariate analysis (*p* = 0.001; *p* = 0.038, respectively). In multivariate analysis, central VF progression was associated with a longer follow-up period, lower mean MOPP, and smaller longest EOBT angular location (*p* = 0.0001; *p* = 0.01; *p* = 0.009, respectively) (Table 6).

**Table 6:** Risk factors associated with visual field progression in the central visual field.

Risk factors	Central visual field progression 55 (36.4%)			
	Univariate analysis		Multivariate analysis	
	OR (95% CI)	*p* value	OR (95% CI)	*p* value
Follow-up period (yrs)	1.139 (1.057, 1.234)	**0.001**	1.185 (1.091, 1.297)	**0.0001**
Age at the first visit (yrs)	0.998 (0.971, 1.025)	0.858		
Male sex	0.699 (0.357, 1.369)	0.296		
Mean number of medications	0.962 (0.563, 1.616)	0.886		
Baseline IOP (mmHg)	1.084 (1.008, 1.175)	**0.038**		
Mean IOP (mmHg)	1.043 (0.934, 1.165)	0.453		
Central corneal thickness (μm)	0.994 (0.984, 1.004)	0.251	0.990 (0.978, 1.001)	0.083
Mean MOPP (mmHg)	0.955 (0.909, 1.0002)	0.057	0.931 (0.879, 0.981)	**0.01**
Axial length (mm)	1.117 (0.891, 1.404)	0.337		
Baseline MD (dB)	0.952 (0.859, 1.054)	0.342	0.911 (0.811, 1.021)	0.11
Maximum EOBT location (°)	0.995 (0.986, 1.003)	0.220	0.986 (0.976, 0.996)	**0.009**
Maximum EOBT length (μm)	1.0004 (0.999, 1.002)	0.451		

*CI* confidence interval,* EOBT* externally oblique border tissue,* IOP* intraocular pressure,* MD* mean deviation,* MOPP* mean ocular perfusion pressure.

The best multiple logistic regression model was chosen based on the Akaike Information Criterion. Factors with statistical significance are marked in bold.

## Discussion

**Figure 2:**
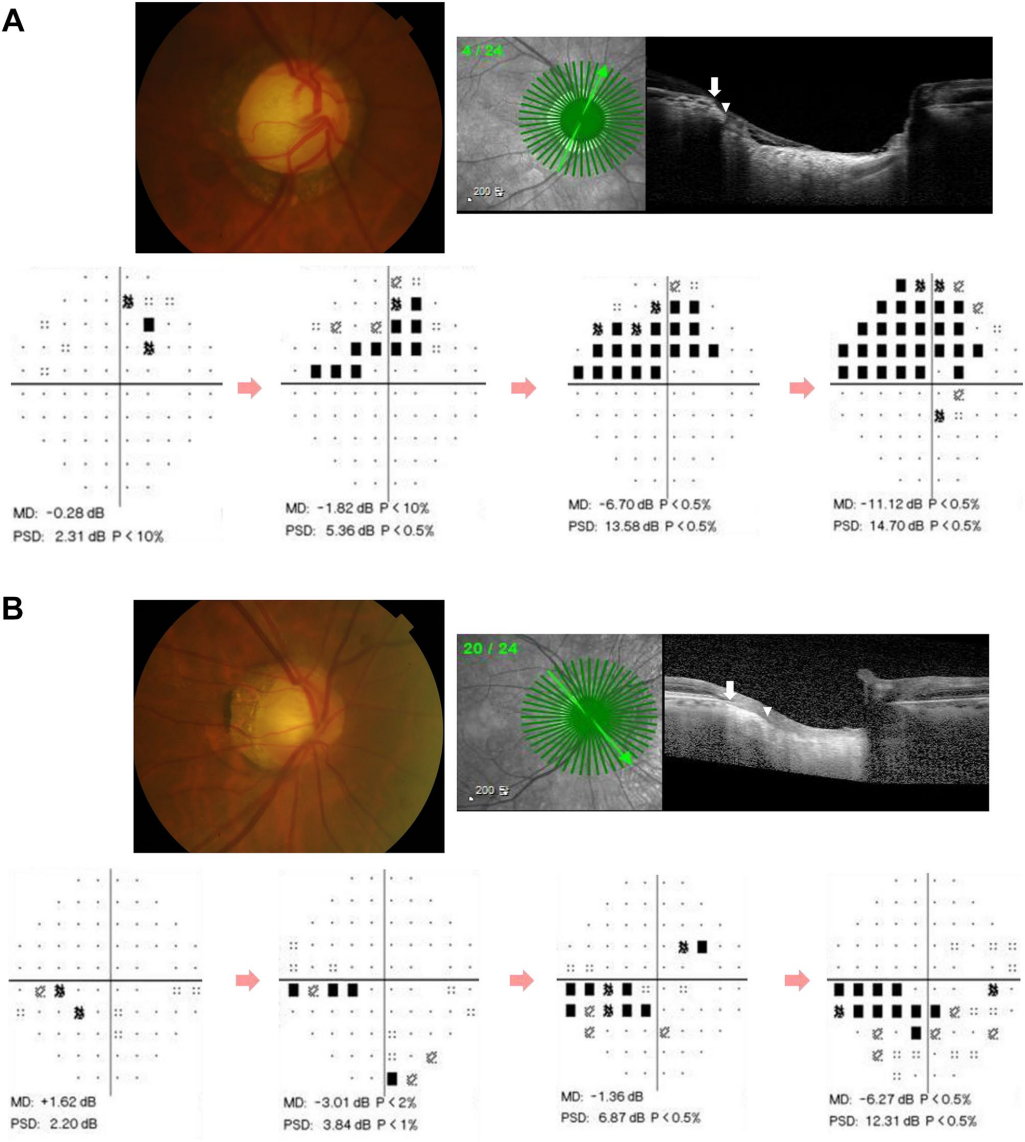
Representative cases showing the relationship between the direction of the maximum externally oblique border tissue (EOBT) and the location of glaucomatous damage. (**A**) Images from a 79-year-old man with primary open-angle glaucoma (spherical equivalent, – 0.75 diopters [D]; axial length, 23.03 mm). The location of the maximum EOBT was + 30°. Mean deviations (MD) on static automated perimetry C30-2 were – 6.30 decibels (dB) on the first exam in 2001 and – 11.12 dB with superior visual field progression on the last exam in 2018. (**B**) Images from a 60-year-old man with NTG with myopia (spherical equivalent, – 3.75 D; axial length, 25.97 mm). MDs were + 1.62 dB on the first exam in 2006 and – 6.27 dB with inferior visual field progression on the last exam in 2017. The location of the maximum EOBT was – 37.5°.

The present study demonstrated that the location of VF progression was significantly associated with the longest EOBT location in OAG eyes (Fig. 2). This result confirms the previous finding that EOBT has a topographical association with glaucomatous damage^14,15^, and further identifies preferential sites of VF progression associated with detailed deep ONH morphology. In addition, other factors including mean MOPP, baseline IOP, and mean IOP also affected specific VF progression patterns in OAG eyes. The present study is the first to demonstrate a possible relationship between the longest EOBT and VF progression patterns in OAG eyes over the long-term period.

Figure 1 clearly shows the main finding of the present study, namely that VF progression pattern varies according to the location of the longest EOBT. Moreover, this relationship was more pronounced when the location of the longest EOBT was far from the macula. If the longest EOBT location was over + 45 degrees (group 5), VF progression occurred mostly in the superior hemifield. In contrast, if the longest EOBT location was at less than -45 degrees (group 1), the initial VF defect was inferiorly dominant at baseline, and further VF progression occurred more in the inferior hemifield than other groups. In other groups, the initial VF defect pattern was relatively superiorly dominant, but further overall VF progression appeared to occur simultaneously in superior and inferior VFs.

In previous studies, relationships between ONH morphology and preferential location of VF progression have been detected^14,17–21^. These previous studies used parameters such as parapapillary atrophy (PPA) or disc tilt as biomarkers indicative of ONH morphology. In the present study, EOBT measured by OCT was used as a biomarker of deformation around the ONH^22^. EOBTs were found frequently, even in ONH without PPA; consistently, the longest EOBT was significantly associated with the VF defect pattern in non-myopic eyes as well as myopic eyes in a previous study^14^.

Nevertheless, the structural characteristics of the EOBT could not completely explain VF progression patterns in OAG patients. For example, VF progression in the superior field was not significantly associated with longest EOBT location. There may be several possible reasons for this. First, most patients already had a damaged superior hemifield defect initially except those in group 1, and further VF progression could have been less substantial in follow-up exams. However, the initial VF defect pattern of each group was associated with ONH deformation, consistent with previous studies^14,15,21^. Second, factors other than EOBT could contribute simultaneously to the VF progression pattern. In the present study, a thinner CCT and lower mean MOPP were associated with superior VF progression. Thinner CCT^23–26^ and lower mean MOPP^9,27–29^ are well known progression risk factors in glaucoma. Worse baseline MD was only associated with inferior VF progression, but not with superior VF progression in the OAG eyes. Considering that superior hemifield was mostly damaged to some extent initially especially when VF had worse MD, a greater chance of VF progression might occur relatively in the inferior VF in the eyes with longer axial length and worse baseline MD.

Lower mean MOPP was associated with central VF progression as well as superior VF progression in the current study. This result is consistent with previous reports that vascular factors including hypotension, migraine, Raynaud phenomenon, blood pressure, and unstable MOPP are associated with central VF defects or progression^8,9,27^. However, no previous study has reported a relationship between lower MOPP and VF progression in the superior hemifield. In previous studies, changes in vascular structures located in superficial and deep ONH layers were usually found in relatively late-stage glaucoma^16,30,31^. Considering that superior VFs consistent with an inferior RNFL defect were present at initial diagnosis in the current study, corresponding vascular structures such as superficial and deep capillaries around the ONH in that area could be damaged more than other areas. We speculate that this could induce more significant VF aggravation if there is lower blood perfusion to those damaged capillaries.

Central VF progression was also associated with higher baseline IOP in OAG eyes. In the previous study, higher IOP was associated with VF progression in overall VF including central 10° hemifield in normal-tension glaucoma^32^.

Meanwhile, we speculated that lower mean IOP might be the result instead of the cause of the overall VF progression. If the eyes showed VF progression, the clinician might add more IOP-lowering medications and eventually induce lower mean IOPs. For the limitation of the study design, this can be hardly proven for now. Further studies to show the causal relations between the factors are warranted.

Glaucoma is defined as a progressive optic neuropathy with unique VF patterns such as nasal step or arcuate scotoma that are usually located in the superior hemifield. The VF pattern is associated with glaucomatous RNFL defects of ONH. Previous studies have shown that geometric features around the ONH such as disc tilt or PPA are associated with initial VF defect patterns in glaucoma eyes with myopia^11,12^. Considering that myopic changes associated with ONH may be related to axial elongation during the growth period, regional structural differences around the optic nerve may also exist in eyes with various axial lengths^22^. Different ONH regional features such as EOBT might confer different susceptibilities of structures around the ONH to the given IOP in the eye. We hypothesized that IOP-related stress and strain around the ONH may induce initial structural damage including lamina cribrosa and RNFL, especially at the most vulnerable region, which corresponds to the longest EOBT. In OAG eyes, different EOBTs are associated with structurally weak areas around the ONH and induce initial glaucomatous damage and further VF progression for a given IOP in tandem with known risk factors such as ocular perfusion pressure.

There are several limitations to this study. First, we evaluated a relatively small number of cases. Thus, in several sub-analyses, some factors were close to but did not reach statistical significance. For compensating this limitation, we analyzed the factors associated with VF progression “including” specific patterns and “only with” specific patterns and tried to find the possible associated factors as many as possible. Second, the present study was designed as a retrospective longitudinal study. Because of the limitations of this study design, it was not possible to determine causal relationships between factors. For example, associated factors such as lower mean IOP can be the cause or the result of the VF progression in central VF. Third, OAG eyes at different stages were included in the analysis. If some region in the VF was already involved to some extent, there may have been less progression in that region. Fourth, The OAG eyes without EOBT were not included in this study. Although, this may arise selection bias, the reason for selecting such group was because an eye without EOBT results in a missing data. The main purpose of this study was to see the correlation between the location of the maximum EOBT and direction of progression of visual field defect. Fifth, the definition of visual field progression used in this study is different from the conventional definition. 42.2% of the patients showed VF progression in this study, which is significantly higher than the literature. If the existing definition is a macroscopic point of view, the definition used in this study was a microscopic point of view that sees changes from the point of view of each point in the visual field map. Therefore, visual field examination findings that were not clinically determined to be progression were considered progression in this study. Sixth, there is an overlap between the subgroups in subgroup analyses such as the central progression group and the superior or the inferior progression groups. However, the reason for this classification is that, the main objective of this study was to compare between the group with VF progression only in the superior VF and the group with VF progression only in the inferior VF. The secondary objective was to find the risk factors of the central VF progression. The central VF defect was further analyzed because it is crucial in clinical practice in view of the fact that it can affect the visual acuity.

In conclusion, the location of the longest EOBT and other factors such as mean MOPP and mean IOP contribute to specific VF progression patterns in OAG eyes.

## Methods

This study was retrospective in nature, and subjects comprised OAG patients who visited Samsung Medical Center (Seoul, Korea) for at least 5-years of follow-up between July 2007 and December 2020. This study followed all guidelines for experimental investigation in human subjects, was approved by the Samsung Medical Center Institutional Review Board (2020-07-141-001), and adhered to the tenets of the Declaration of Helsinki. Informed consent was waived by the Samsung Medical Center Institutional Review Board.

Inclusion criteria were a diagnosis of OAG in addition to (1) the existence of externally oblique border tissue on enhanced depth imaging (EDI) spectral domain (SD)-OCT scans and (2) mean deviation ≥ − 12 dB on the first VF test. Exclusion criteria were concomitant ocular or systemic diseases that could affect VF tests, such as a history of vision-threatening retinal disease (e.g., retinal detachment, retinal vein occlusion) or neurologic disease. If glaucoma existed bilaterally, one of the two eyes was randomly included in the study.

Each participant underwent a comprehensive ophthalmic examination, including slit-lamp biomicroscopy, Goldmann applanation tonometry, gonioscopic examination, dilated stereoscopic examination of the ONH, color and red-free fundus photography (TRC-50DX model; Topcon Medical System, Inc., Oakland, NJ, USA), automated perimetry using a central 30–2 Humphrey field analyzer (HFA model 740; Humphrey Instruments, Inc., San Leandro, CA, USA) with the Swedish interactive threshold algorithm standard, axial length measurement (IOL Master®; Carl Zeiss Meditec, Jena, Germany), and ultrasonographic pachymetry (Tomey SP-3000; Tomey Ltd., Nagoya, Japan). The extent of the VF defect was measured using MD and PSD. VF tests were repeated once or twice a year, depending on the patient’s condition. Reliable VF analysis was defined as a false-negative rate of < 15%, a false-positive rate of < 15%, and a fixation loss of < 20%. Unreliable VF test results were excluded from the analyses.

IOP at baseline was defined as the IOP at the first visit. The number of IOP-lowering medications at first visit was counted according to type (e.g., if a patient used a fixed combination, the patient was considered to be taking two medications). SBP and DBP were measured once at the first visit. MOPP was obtained using the formula MOPP = 2/3 [DBP + 1/3 (SBP—DBP)]—IOP.

### Classification of progression pattern of the visual field defect

**Figure 3:**
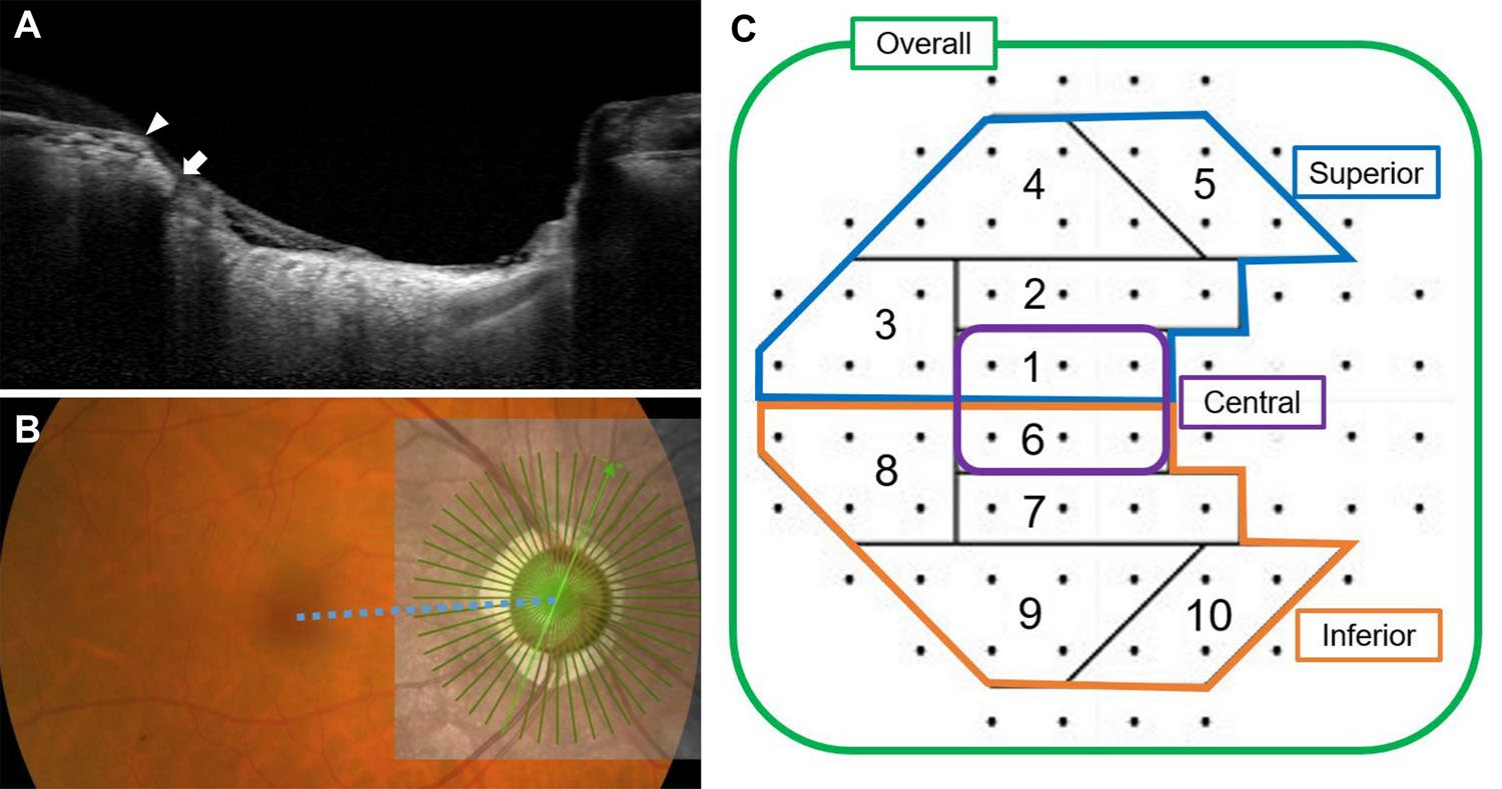
Methods for measuring the length and angular location of the longest externally oblique border tissue (EOBT) and classification of visual field test points. (**A**) Cross-sectional image of the optic nerve head. EOBT length was measured as the distance between Bruch’s membrane opening (BMO) (arrowhead) and the inner end of the border tissue (arrow). (**B**) Enhanced depth imaging optical coherence tomography (EDI-OCT) scan was obtained using 48 radial line B-scans (each at an angle of 3.75°). The OCT image was superimposed on the fundus photo of the same eye. The angular location of the maximum EOBT was defined as the angle between the fovea-BMO axis (dotted blue line) and the reference line (light green line with an arrowhead) obtained while measuring the maximum EOBT length. (**C**) Ten visual field sectors based on anatomical relationships between visual field test points in the Humphrey 30–2 test and location of retinal nerve fiber layer bundles. Sectors 1 and 6 were regarded as the central zone. Five upper sectors—sectors 1 to 5—and five lower sectors—sectors 6 to 10—were regarded as the superior zone and inferior zone, respectively.

For each patient, we calculated the rate of VF loss (decibels per year) at each test location of the total deviation plot. Test locations were categorized into 10 zones according to the normal anatomy of the retinal nerve fiber layer^33^. Test points that can show high variability due to lens rim artifacts or proximity to physiological scotoma were excluded. Therefore, a total of 44 points were included in the study (Fig. 3). We considered a rate of deterioration faster than − 1 dB/year with sensitivity at the 1% level in any one point in zones 1 and 6 or any two points in the other eight zones as clinically significant deterioration based on the modification of the result of the previous report^34^. If significant deterioration was detected in zones 1 and 6, it was classified as central VF progression. Superior VF progression was defined when the VF progression pattern included significant deterioration in zones 1 to 5, and inferior VF progression was defined when the VF progression pattern included significant deterioration in zones 6 to 10. For clear comparison, 16 cases with progression in both superior and inferior zones were excluded.

### Measurement of the length and location of the longest externally oblique border tissue

Measurement of EOBT length by EDI SD-OCT was performed on each patient according to methods described in detail in a previous study^16^. Forty-eight radial-line B-scans (each at an angle of 3.75°) centered on the optic disc were obtained and every other section (each at an angle of 7.5°) was used. We defined the length of the EOBT as the distance between the inner end point of the EOBT and Bruch’s membrane opening (Fig. 3). If the OCT image quality was too poor to recognize BMO or border tissue, the next scan section was used. The length of EOBT was measured in each scan and the scan with the longest EOBT was selected. A fundus photo of the same eye was superimposed on the OCT scan to identify the location of the fovea. The line connecting the center of the BMO and the fovea was defined as the fovea-BMO axis. The angle between the radial line and the fovea-BMO axis was defined as the angular location of the maximum EOBT. If a radial line was located below the fovea-BMO axis, it was regarded as a positive angle. Otherwise, it was regarded as negative angle.

**Figure 4:**
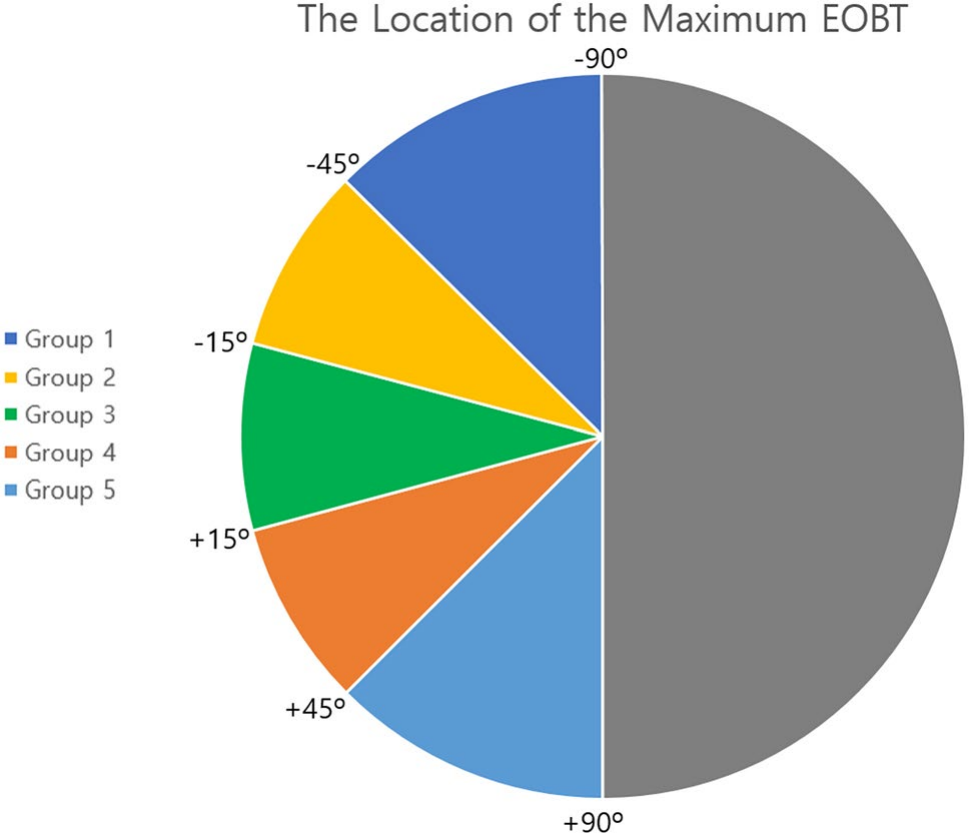
Classification for subgroup analysis according to the angular location of the longest externally oblique border tissue (EOBT). Since this figure shows the angular location with respect to the right eye, in the case of the left eye, the angular location was indicated by flipping it horizontally. Group 1: < – 45°, Group 2: – 45° ~ – 15°, Group 3: – 15° ~  + 15°, Group 4: + 15° ~  + 45°, Group 5: >  + 45°.

We divided patients into five arbitrary groups based on the angular location of maximum EOBT length as follows: Group 1: < − 45°, Group 2: − 45° ~ − 15° Group 3: − 15° ~  + 15°, Group 4: + 15° ~  + 45°, Group 5: >  + 45° (Fig. 4).

Two independent observers (HJK and EDS) measured the length and location of the longest EOBT independently. The average length and location of the longest EOBT were used in subsequent analyses.

### Statistical analysis

Statistical analyses were performed using R software v3.6.3 (R Foundation for Statistical Computing, Vienna, Austria). Kruskal–Wallis test and Fisher’s exact test were used to compare means and homogeneity of the clinical variables among the five groups classified by angular location of the longest EOBT. Univariate and multivariate logistic regression analyses were used to calculate the odds ratios (ORs) of clinical variables for each dominant VF progression pattern. The ORs for the group with VF progression at each field were against the group without VF progression. In multivariate analysis, stepwise variable selection procedures based on the Akaike Information Criterion were implemented. ORs are presented as means with 95% confidence intervals. Two-sided *P* values are reported for all tests; *P* values less than 0.05 were considered statistically significant.

## Data Availability

The datasets that were analyzed for this study are available from the corresponding author on a reasonable request.
